# Supplementary material for: Conjugated cross-linked phosphine as broadband light or sunlight-driven photocatalyst for large-scale atom transfer radical polymerization
Source: Nat Commun. 2023 May 20;14:2891. doi: 10.1038/s41467-023-38402-y (PMC10199896; doi:10.1038/s41467-023-38402-y)
Supplement: Supplementary file 1 — Supplementary Information [file 41467_2023_38402_MOESM1_ESM.pdf]

Supplementary Information

Conjugated Cross-linked Phosphine as Broadband Light or Sunlight-Driven Photocatalyst for Large-Scale Atom Transfer Radical Polymerization

Wei-Wei Fang[1†], Gui-Yu Yang[1†], Zi-Hui Fan[1], Zi-Chao Chen[1], Xun-Liang Hu[2], Zhen Zhan[2], Irshad Hussain[3], Yang Lu[1], Tao He[1]*, Bi-En Tan[2]*

* Corresponding author. Email: taohe@hfut.edu.cn (T. He); bien.tan@mail.hust.edu.cn (B. E. Tan)

This PDF file includes:

Materials and Methods
Supplementary Figure. 1 to 35
Supplementary Table 1 to 12
Supplementary References

Supplementary Discussion

Materials and Methods

General Information

Methyl acrylate (MA; Aladdin, 99%), n-butyl acrylate (n-BA; Aladdin, 99%), (2-methoxyethyl) acrylate (MEA; Aladdin, >98.0%), 2,2,2-trifluoroethyl acrylate (TFEA; Aladdin, >98.0%), benzyl acrylate (BzA; Aladdin, 97.0%), tert-butyl acrylate (t-BA; Aladdin, 99%), methyl methacrylate (MMA; Aladdin, 99%), n-butyl methacrylate (n-BMA; Aladdin, 99%), cyclohexyl methacrylate (CHMA; Aldrich, 97%), lauryl methacrylate (LMA; Aladdin, 96%), poly(ethylene glycol) methyl ether methacrylate (OEGMA; Aldrich), and styrene (St; Aladdin, 99%) were passed through a column of basic alumina to remove polymerization inhibitor prior to use. Triphenylphosphine (PPh_3; Aladdin, >99.0%), 1,4-dimethoxybenzene (DMB; Aladdin, 99%), anhydrous iron(III) chloride ($FeCl_3$; Aladdin, >99.99%), ethyl α-bromoisobutyrate (EBiB; Aladdin, 98%), ethyl α-bromophenylacetate (EBPA; Aladdin, >98%), 2-hydroxyethyl 2-bromoisobutyrate (HBiB; Aldrich, 95%), poly(ethylene glycol) bis(2-bromoisobutyrate) (M_n = 4300, Br-PEG-Br; Aldrich), tris[2-(dimethylamino)ethyl]amine (Me_6TREN; Aladdin, >98%), tris(2-pyridylmethyl)amine (TPMA; Aladdin, >98.0%), N,N,N′,N″,N″-pentamethyldiethylenetriamine (PMDETA; Aladdin, 99%), triethanolamine (TEOA; Aladdin, >99.0%), copper(II) bromide ($CuBr_2$; Aladdin, 99%), sodium bromide (NaBr, Aladdin, 99%), nitrobenzene, dimethyl sulfoxide (DMSO), N,N-dimethylformamide (DMF), and acetonitrile (MeCN) were used as received.

^{1}H nuclear magnetic resonance (^{1}H NMR) and diffusion ordered spectroscopy nuclear magnetic resonance (DOSY NMR) measurements were performed on an Angilent VSR 600 MHz spectrometer. Solid-state ^{13}C cross polarization/magic angle spinning (CP/MAS) and ^{31}P NMR spectra were performed on a JNM-ECZ600R (JEOL RESONANCE Inc., Japan) 600 MHz spectrometer. The experiments were

carried out on a 3.2 mm resonance probe with a spinning rate of 12 kHz. Molecular weight properties of the polymers were determined by size exclusion chromatography (SEC). The SEC instrument was equipped with a Waters 1515 pump and a Waters 2414 differential refractometer using PSS columns (SDV 10000, 2500, and 100 Å) with tetrahydrofuran (THF) as eluent at 35 °C and a flow rate of 1 mL min^{-1}. Linear polystyrene standards were used for calibration. The surface areas were measured by CO_2 adsorption and desorption at 195 K using a Micrometrics ASAP 2020 volumetric adsorption analyzer. Powder samples were degassed at 110 °C for 8 h under vacuum (10^{-5} bar) before analysis. Thermogravimetric (TG) analysis was performed on a NETZSCH STA449 F5 by heating samples at 10 °C min^{-1} under air to 800 °C. Scanning electron microscope (SEM) was performed for microstructural investigation and conducted on a Merlin Compact field emission scanning electron microscope. Constituent elements of the samples were analyzed by energy dispersive spectroscopy (EDS, X-Max, Oxford). Transmission electron microscopy (TEM) was performed on a Hitachi HT7700. Diffuse reflectance ultraviolet/visible/near infrared (UV-Vis-NIR) spectrum was acquired using an Angilent CARY 5000 spectrophotometer. Electrosprayionization mass spectrometry (ESI-MS) analysis was performed at Thermo Scientific Vanquish Q Exactive Plus mass spectrometry. UV-Vis absorption spectra of solution were measured on a Shimadzu UV2600 UV-Vis spectrometer. The photoluminescence (PL) spectra of the photocatalysts were studied by a Shimadzu RF-6000 3D fluorescence spectrophotometer. Fluorescence quantum yield and their relation to lifetimes of photocatalysts were conducted on an Edinburgh FLS 1000. X-ray photoelectron spectroscopy (XPS) was carried out on a Thermo ESCALAB 250Xi instrument. Laser particle size analysis was conducted on a Malvern Mastersizer 2000. Zeta Potential was recorded on a Malvern Zetasizer Nano ZS90. The photocatalytic performance was measured under the irradiation of visible light (>420 nm) at 15 A current with 300 W Xe lamp (Beijing Perfect Light Co. Ltd, PLS-SXE300). The diameter of the photoreactor

was 7.8 cm. The whole photocatalytic process was kept at room temperature (25 °C) with a light intensity of 130 mW cm^{-2}. The blue (λ_{max} = 455 nm), green (λ_{max} = 540 nm), orange (λ_{max} = 590 nm), red (λ_{max} = 630 nm), and white LED lamps were purchased from NVC Lighting. The LED strips were mounted inside a glass container (diameter = 15 cm, height = 7.5 cm) and a cooling fan was used during polymerization to maintain the reactions at room temperature, the optical power density was regulated by a sliding resistor. Far red1 (λ_{max} = 730 nm), far red2 (λ_{max} = 760 nm), NIR1 (λ_{max} = 800 nm), NIR2 (λ_{max} = 850 nm), and NIR3 (λ_{max} = 940 nm) LED lamps were purchased from Shenzhen Xusheng Electronic Technology Co., Ltd. The intensities of light sources were measured using TES-1333R. The emission spectra of LED light sources were measured using HP-350C (380-780 nm) or HP-350IR (550-1100 nm) illuminometer purchased from Hangzhou LCE Intelligent Detection Instrument Co., Ltd.

Polymerization Procedures

General procedure for temporal control in photoinduced ATRP of MA using PPh_3-CHCP under green light irradiation

The photocatalyst PPh_3-CHCP (1.6 mg), MA (1.62 mL, 18.0 mmol, 200 equiv.), DMF (1.62 mL), a stock solution of $CuBr_2$ (0.8 mg, 3.6 μmol, 0.04 equiv.), and Me_6TREN (4.8 μL, 18.0 μmol, 0.2 equiv.) in DMF (40 μL) were added to a Schlenk tube under nitrogen atmosphere. The tube equipped with a magnet bar was sealed with a rubber septum and degassed by three freeze-vacuum-thaw cycles. A 13.2 μL aliquot of EBiB (90.0 μmol, 1 equiv.) was introduced into the tube *via* syringe. The tube was irradiated under green LEDs to start the polymerization. After 6 h exposure, the reaction tube was kept in dark for 2 h and exposed to repeated cycles for 2 h. In these subsequent intervals, 0.2 mL of reaction mixture were syringed out from the polymerization media and precipitated in methanol. Samples were analyzed by ^{1}H NMR and SEC to determine the monomer conversion and molecular weight properties, respectively.

Procedure for photoinduced ATRP of methyl acrylates using PPh_3-CHCP under green light irradiation

Photoinduced ATRP process of different methyl acrylates using PPh_3-CHCP were similar, except the polymerization of OEGMA was conducted in NaBr aqueous solution. Typical procedure for ATRP of MMA was as follows: the photocatalyst PPh_3-CHCP (0.9-1.9 mg), MMA (0.955 mL, 9.0 mmol, 200 equiv.), DMSO (0.955 mL), TEOA (4.0 mg, 27 μmol, 0.6 equiv.), and a stock solution of $CuBr_2$ (0.4 mg, 1.8 μmol, 0.04 equiv.), and TPMA (2.6 mg, 9.0 μmol, 0.2 equiv.) in DMSO (20 μL) were added to a Schlenk tube under nitrogen atmosphere. The tube equipped with a magnet bar was sealed with a rubber septum and degassed by three freeze-vacuum-thaw cycles. A 7.9 μL aliquot of EBPA (45.0 μmol, 1 equiv.) was introduced into the tube via syringe. The tube was irradiated under green LEDs to start the polymerization. Samples were taken periodically and analyzed by ^{1}H NMR and SEC to determine the monomer conversion and molecular weight properties, respectively. PMMA could be obtained after filtration of PPh_3-CHCP followed by precipitation in methanol directly.

Typical procedure for recycling and reuse of PPh_3-CHCP photocatalyst

Typical procedure was as follows: the polymerization mixture was diluted by adding 20 mL of MeCN and the photocatalyst was separated by centrifugation. The photocatalyst was thoroughly washed with MeCN and separated by centrifugation 3 times and dried before use in the next cycle. A fresh solution of $CuBr_2$/Me_6TREN was used in each cycle.

General procedure NIR photoinduced ATRP penetration experiment using A4 paper as barrier

Typical procedure for NIR photoinduced ATRP penetration experiment using A4 paper as barrier was as follows: the photocatalyst PPh_3-CHCP (3.2 mg), MA (810 μL, 9.0 mmol, 200 equiv.), DMSO (810 μL), and a stock solution of $CuBr_2$ (0.2 mg, 0.9 μmol, 0.02 equiv.), and Me_6TREN (2.4 μL, 9.0 μmol, 0.2 equiv.) in DMSO (20 μL) were added to a Schlenk tube under nitrogen atmosphere. The tube equipped with a magnet bar was sealed with a rubber septum and degassed by three freeze-vacuum-thaw

cycles. A 6.6 μL aliquot of EBiB (45.0 μmol, 1 equiv.) was introduced into the tube via syringe. The tube was exposed to 940 nm LEDs (30 mW/cm^2) with 0.1 mm paper (1 A4 paper) as the barrier for 6 h. ^{1}H NMR and SEC to determine the monomer conversion and molecular weight properties respectively. PMA can be obtained after filtration of PPh_3-CHCP followed by precipitation in methanol directly.

Procedure for large scale polymerization of MMA using sunlight

Typical procedure was as follows: a 250 mL bottle was charged with a stirbar and the catalyst (100 mg) was transferred into a nitrogen-atmosphere glovebox. DMSO (100 mL), MMA (100 mL, 0.955 mol, 200 equiv.), EBPA (0.835 mL, 4.8 mmol, 1 equiv.), TEOA (432 mg, 2.9 mmol, 0.6 equiv.) and a stock solution of $CuBr_2$ (43 mg, 0.192 mmol, 0.04 equiv.), and TPMA (277 mg, 0.955 mmol, 0.2 equiv.) in DMSO (2 mL) were added sequentially *via* pipette. The bottle was then removed from the glovebox, sealed with electrical tape, and placed on the roof of ShengHua Build (7-floored), Hefei University of Technology for 6h. PMMA could be obtained after filtration of PPh_3-CHCP followed by precipitation in methanol directly.

Control Experiments

The photocatalyst PPh_3-CHCP (10 mg) and DMSO (2 mL) were added to a Schlenk tube under a nitrogen atmosphere. The tube equipped with a magnet bar was sealed with a rubber septum and degassed by three freeze-vacuum-thaw cycles. A 15.8 μL aliquot of EBPA (90.0 μmol) and a 13.2 μL aliquot of EBiB (90.0 μmol) were introduced into the tube via syringe. The tube was irradiated under green LEDs. The mixture was diluted by adding 20 mL of MeCN and the photocatalyst was separated by passing through filtrate paper. The residue was concentrated under hyper vacuum to remove solvent and the crude products were analyzed by ESI-MS.

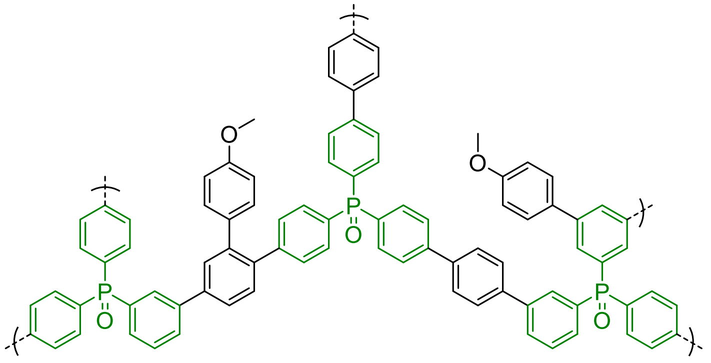

**Supplementary Figure 1 | ** A proposed structure of the PPh_3-CHCP.

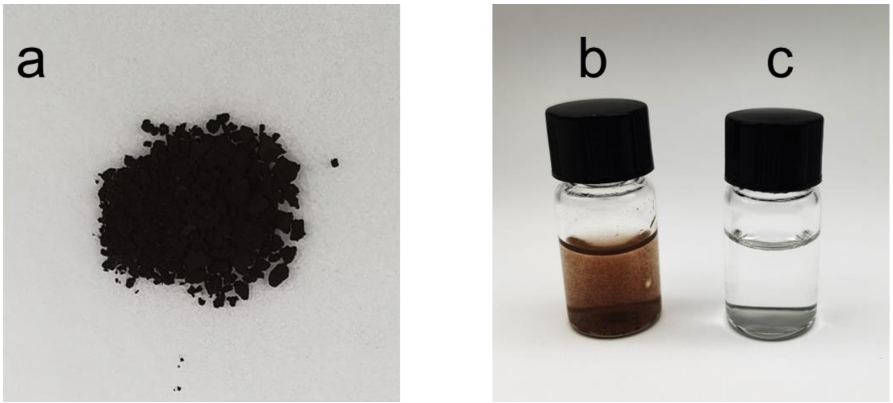

Supplementary Figure 2 | Pictures of PPh_3-CHCP in (**a**) powder form, (**b**) dispersed in MeCN, and (**c**) after filtration of the dispersion in MeCN through syringe filters respectively.

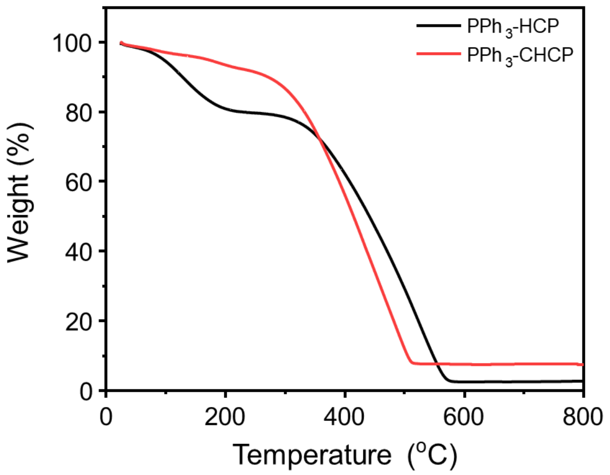

**Supplementary Figure 3 | ** TG curves of PPh_3-HCP and PPh_3-CHCP respectively.

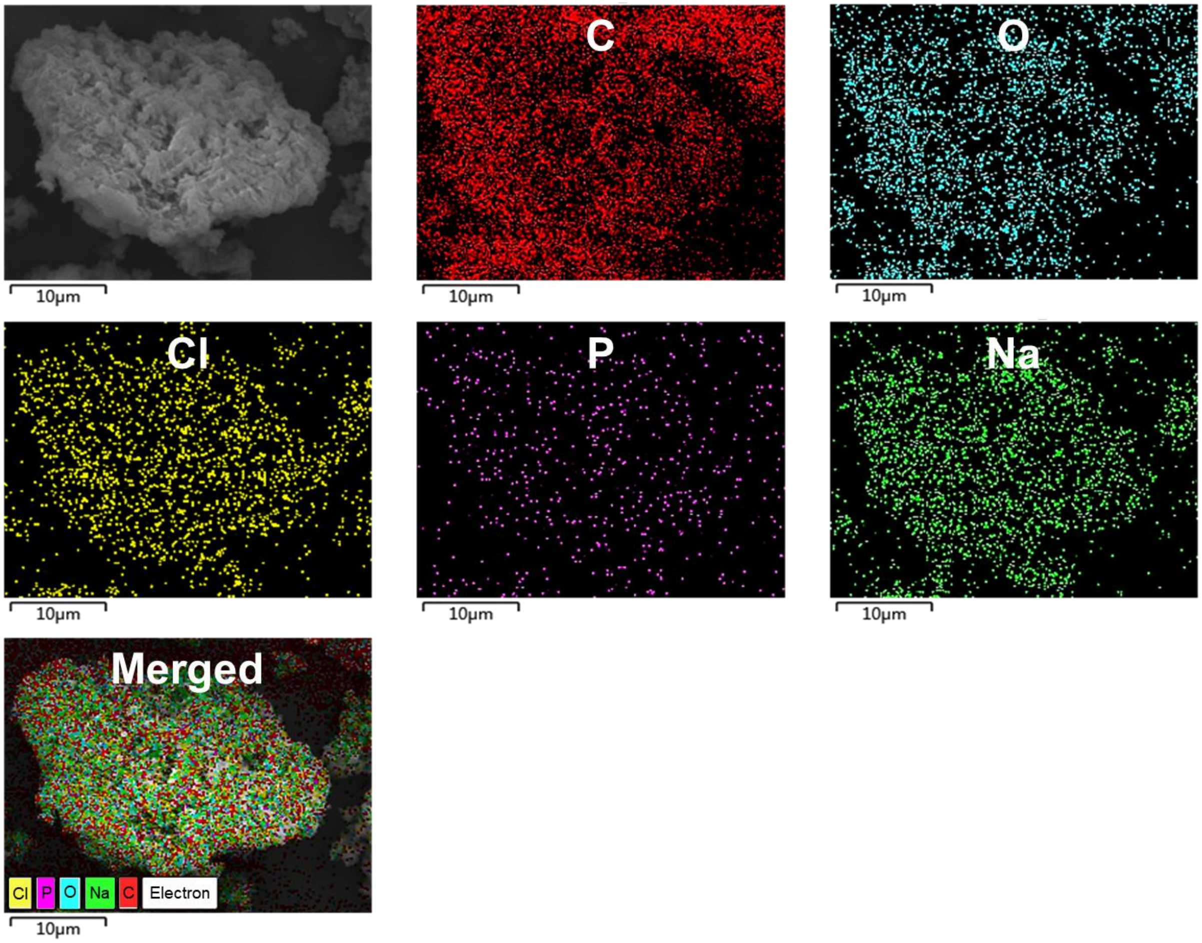

Supplementary Figure 4 | SEM-EDS mapping of PPh_3-CHCP.

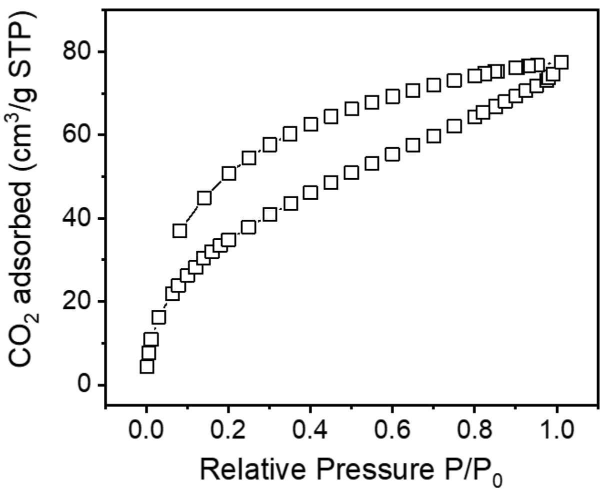

Supplementary Figure 5 | CO_2 adsorption and desorption isotherms of the PPh_3-CHCP.

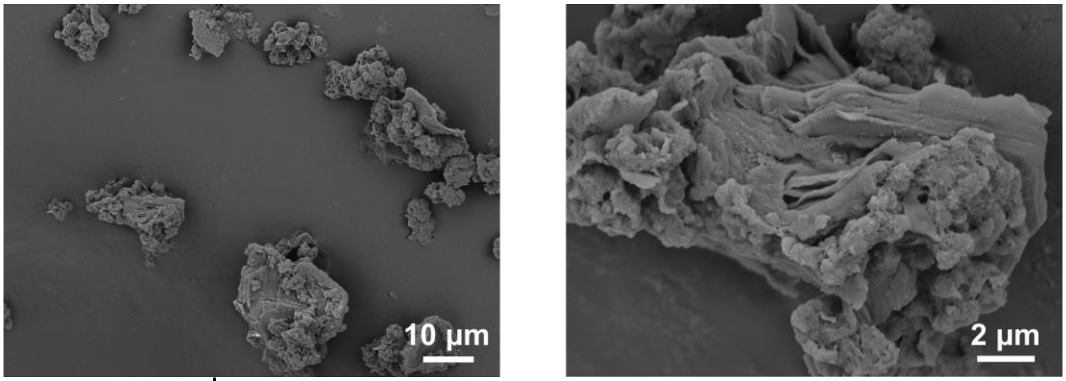

Supplementary Figure 6 | SEM images of the PPh_3-CHCP photocatalyst.

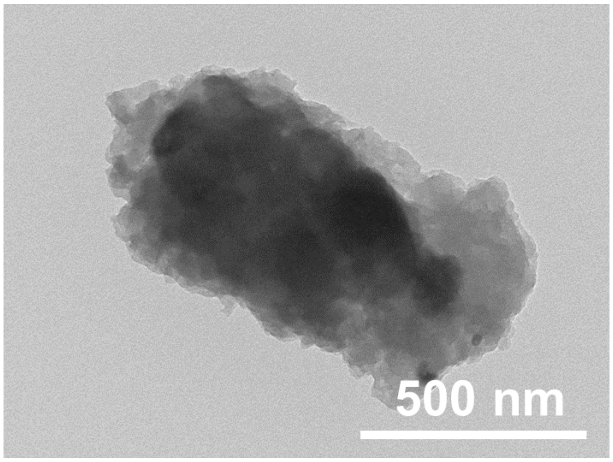

Supplementary Figure 7 | TEM image of the PPh_3-CHCP photocatalyst.

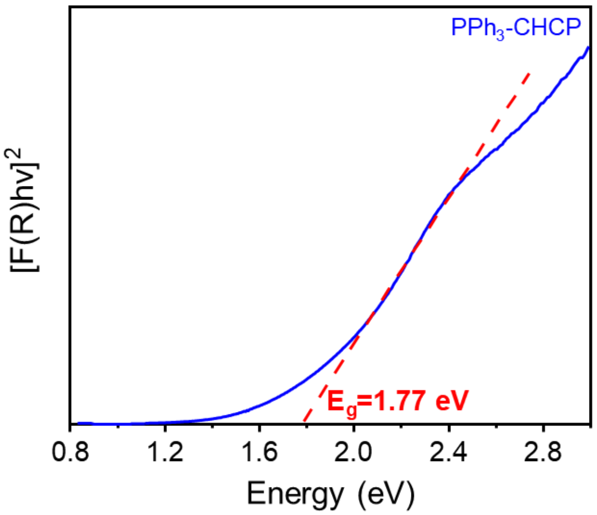

Supplementary Figure 8 | Tauc plot of transformed Kubelka-Munk reflectance as a function of the energy for PPh_3-CHCP.

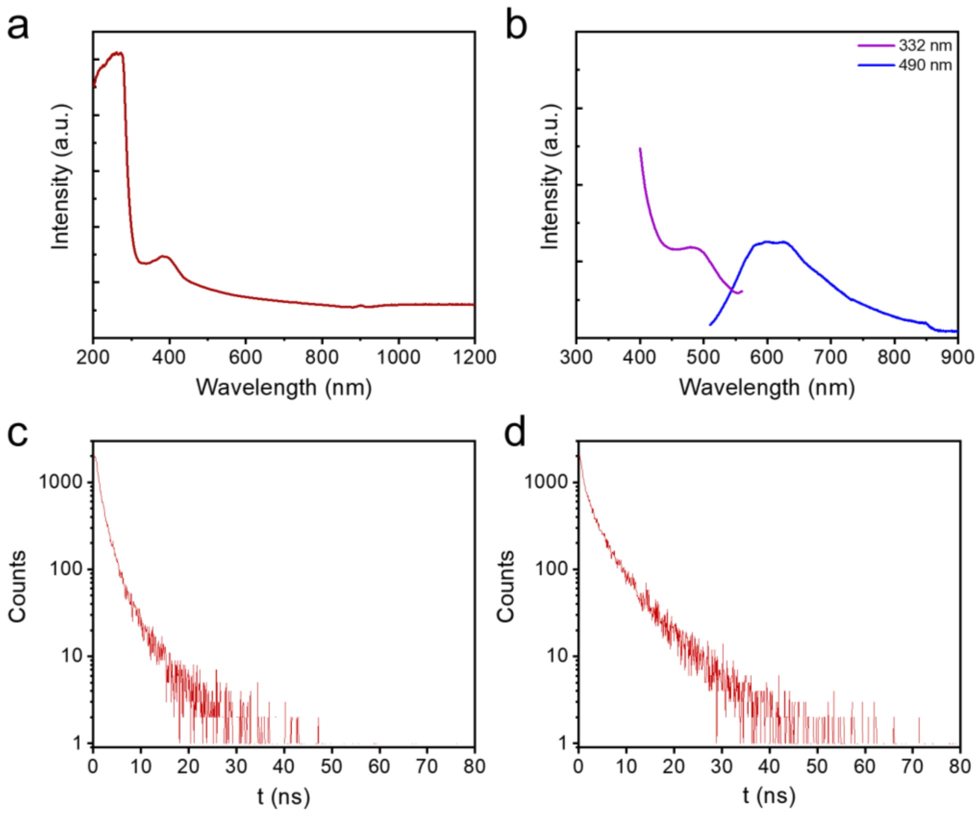

Supplementary Figure 9 | (**a**) UV-Vis absorption spectra of PPh_3-CHCP in DMSO solution. (**b**) Fluorescence spectra of PPh_3-CHCP at the excitation wavelengths of 332 nm and 490 nm, respectively. (**c**) and (**d**) Fluorescence decay curves of PPh_3-CHCP in DMSO solution applying 332 and 490 nm excitation respectively.

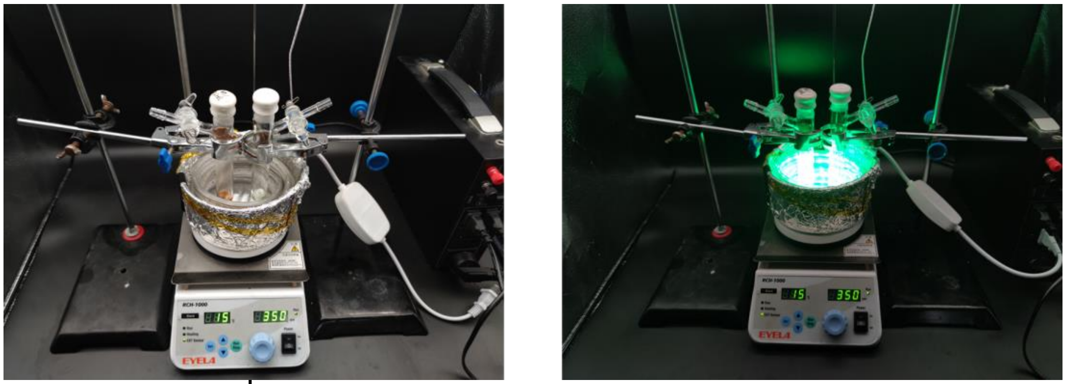

Supplementary Figure 10 | Photoreactor setup used in polymerization reactions. Picture of the photoreactor with green LED strips installed inside a glass container, polymerization reactions with (right vial) and without (left vial) the PPh_3-CHCP photocatalyst.

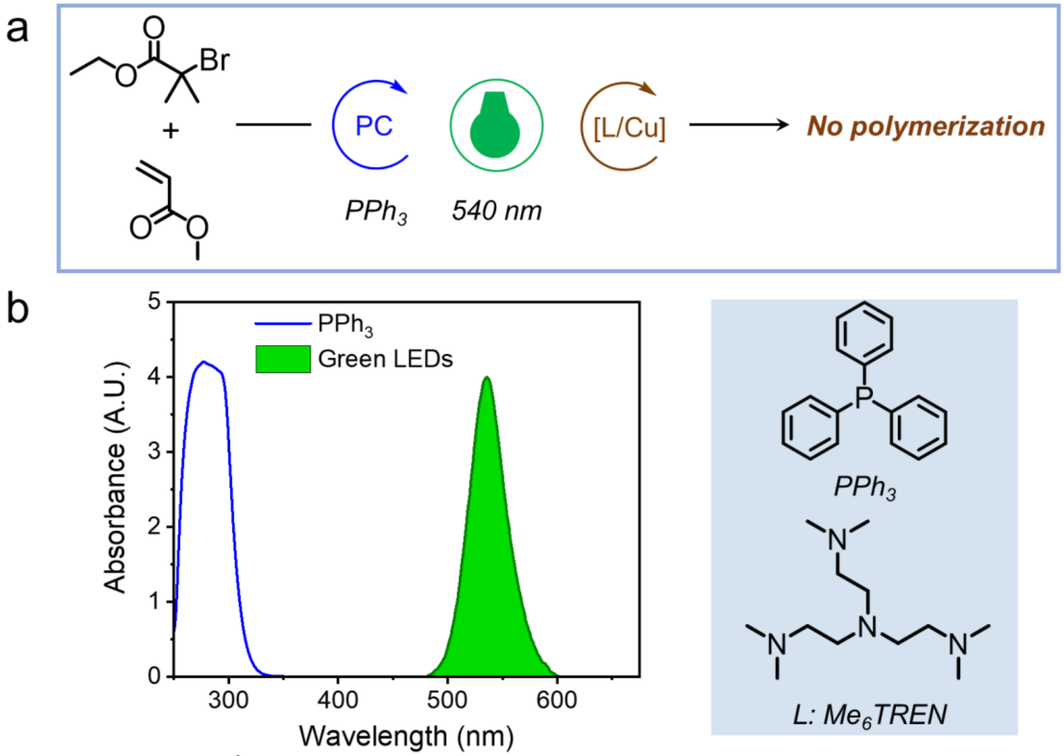

Supplementary Figure 11 | (a) Control experiment in the presence of PPh_3 as a photocatalyst that resulted in no polymerization of MA under green light irradiation. (**b**) UV-Vis absorption spectra of PPh_3 show absorbance in the UV region <400 nm with no overlap with the emission spectra of the green LEDs ($[PPh_3]$ = 1.9 mM in DMSO). Reaction conditions: $[MA]/[EBiB]/[CuBr_2]/[Me_6TREN]$ = 200/1/0.04/0.12 in 50 vol% DMSO, PPh_3 =0.5 or 1 mg/mL (1.9 or 3.8 mM, respectively), irradiated under green light (0.9 mW/cm^2) for 24 h.

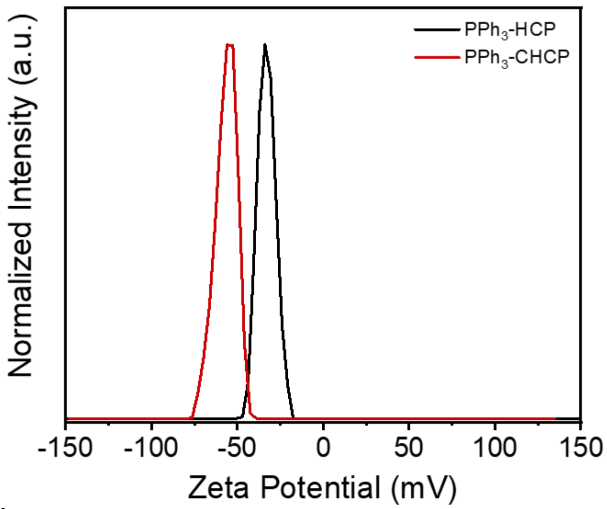

Supplementary Figure 12 | Zeta Potential of PPh_3-HCP and PPh_3-CHCP respectively.

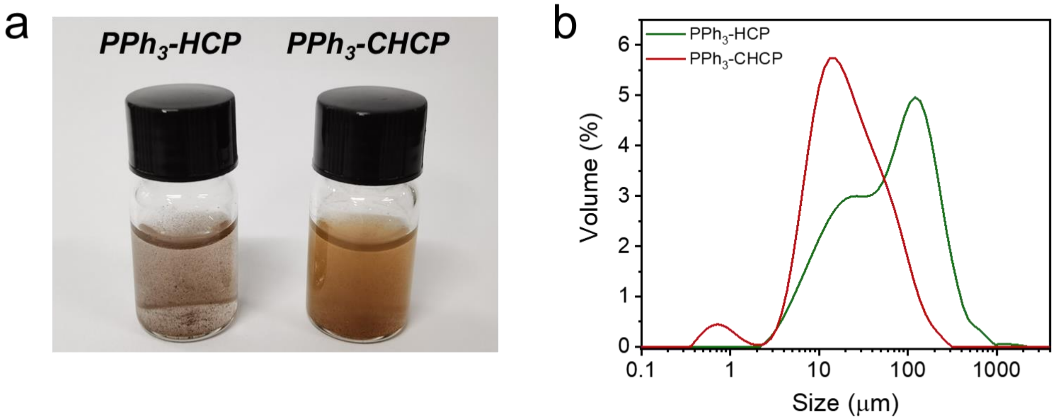

Supplementary Figure 13 | (a) Images of PPh_3-HCP and PPh_3-CHCP dispersed in water respectively. (**b**) Size distribution of PPh_3-HCP and PPh_3-CHCP measured by laser particle analyzer respectively.

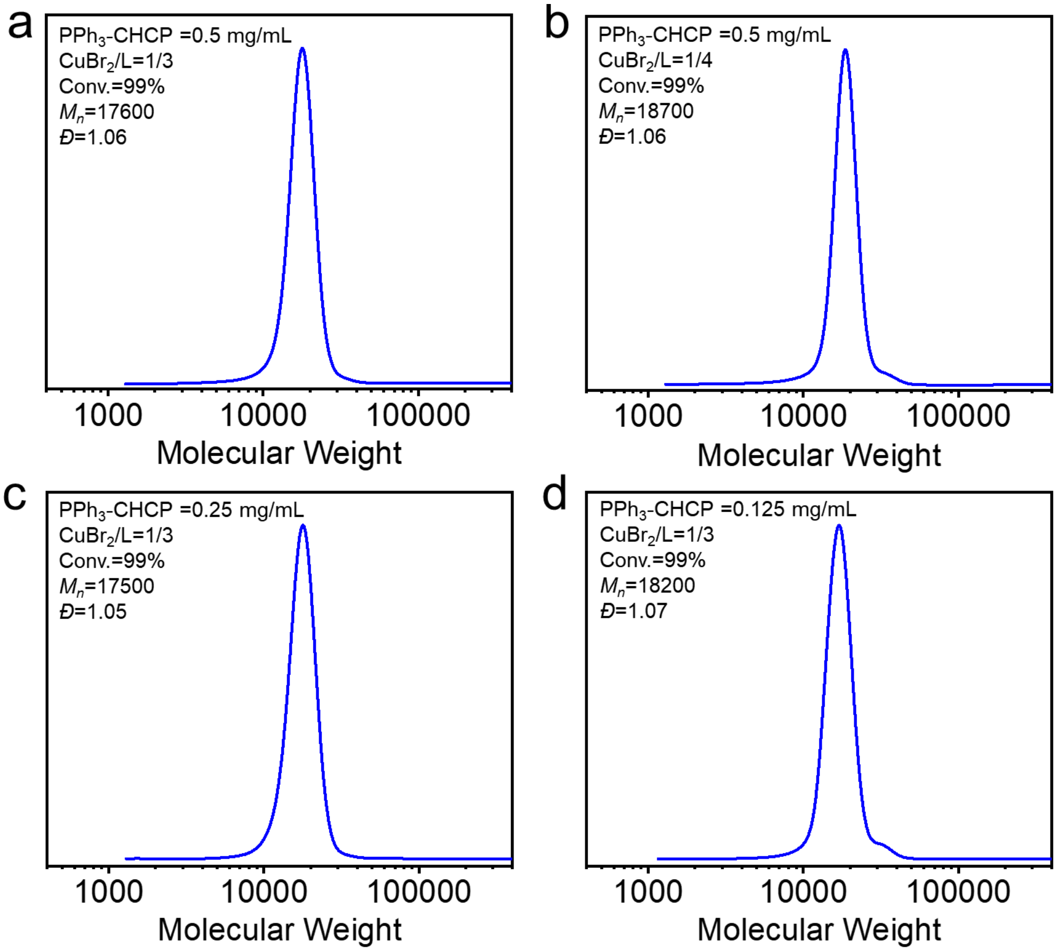

Supplementary Figure 14 | SEC traces of PMA synthesized using PPh_3-CHCP as a heterogeneous photocatalyst in the presence of increasing concentration of the excess ligand. Reaction conditions: [MA]/[EBiB]/[$CuBr_2$]/[Me_6TREN] = 200/1/0.04/x in DMSO (50 vol%), x = 0.12 (**a**) or 0.16 (**b**), PPh_3-CHCP = 0.5 mg/mL, irradiated under green light (0.9 mW/cm^2). SEC traces of PMA synthesized using (**c**) 0.25 and (**d**) 0.125 mg/mL PPh_3-CHCP photocatalyst in the presence of Me_6TREN ligands respectively. Reaction conditions: [MA]/[EBiB]/[$CuBr_2$]/[Me_6TREN] = 200/1/0.04/0.12 in DMSO (50 vol%), irradiated under green light (0.9 mW/cm^2).

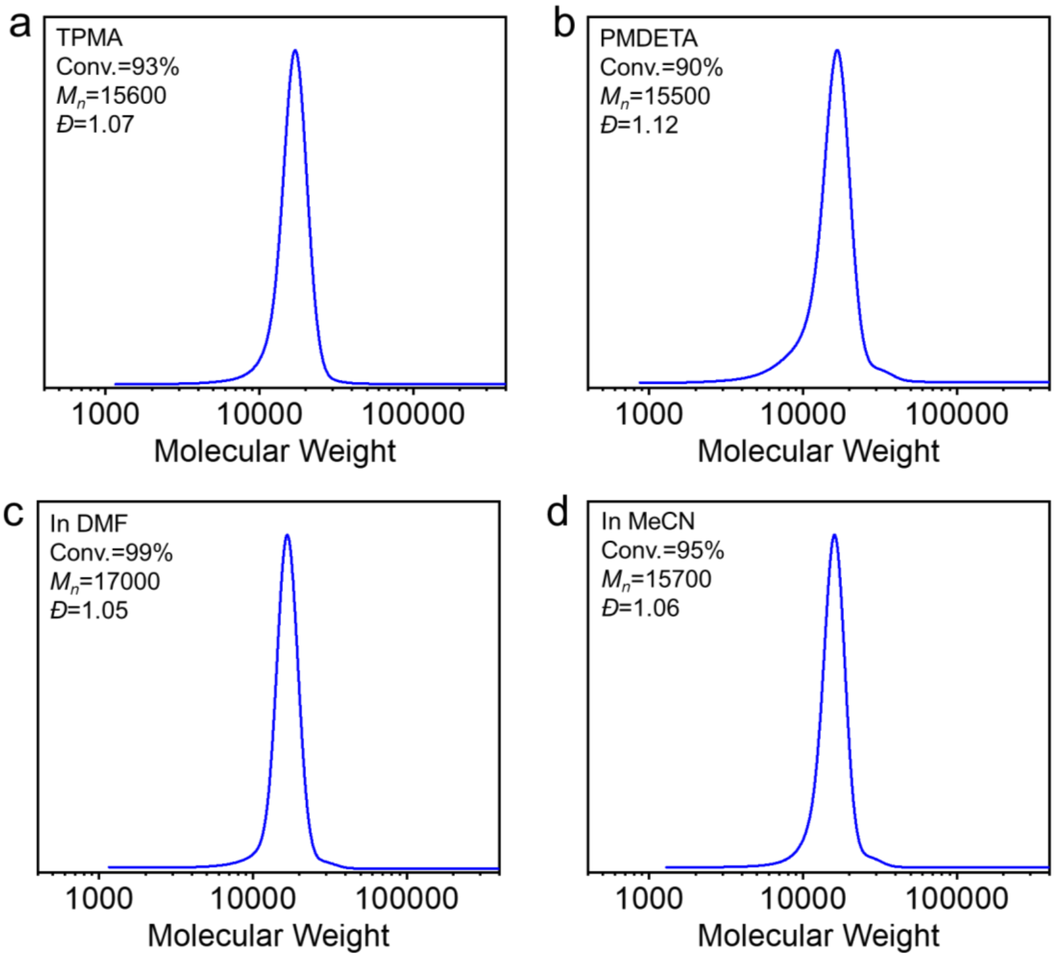

Supplementary Figure 15 | SEC traces of PMA synthesized using PPh_3-CHCP as a heterogeneous photocatalyst in the presence of (**a**) TPMA or (**b**) PMDETA, respectively. Reaction conditions: [MA]/[EBiB]/[$CuBr_2$]/[L] = 200/1/0.04/0.2 in DMSO (50 vol%), irradiated under green light (0.9 mW/cm^2), PPh_3-CHCP = 0.5 or 1 mg/mL for TPMA or PMDETA, respectively. SEC traces of PMA synthesized using PPh_3-CHCP as heterogeneous photocatalyst in (**c**) DMF or (**d**) MeCN, respectively. Reaction conditions: [MA]/[EBiB]/[$CuBr_2$]/[Me_6TREN] = 200/1/0.04/0.2 in DMF or MeCN (50 vol%), PPh_3-CHCP = 2 mg/mL irradiated under green light (0.9 mW/cm^2) for 20 h or 24 h respectively.

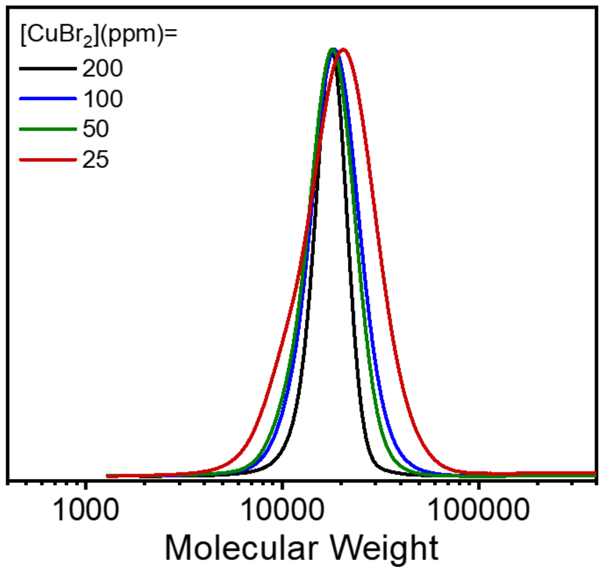

Supplementary Figure 16 | SEC traces of PMA synthesized in the presence of decreasing concentration of the Cu catalyst with Me_6TREN ligand respectively. Reaction conditions: [MA]/[EBiB]/[$CuBr_2$]/[Me_6TREN] = 200/1/*x*/0.12 (*x* = 0.04, 0.02, 0.01, and 0.005 equiv. with respect to initiator corresponding to 200, 100, 50, and 25 ppm with respect to monomer) in DMSO (50 vol%) under green light irradiation (0.9 mW/cm^2).

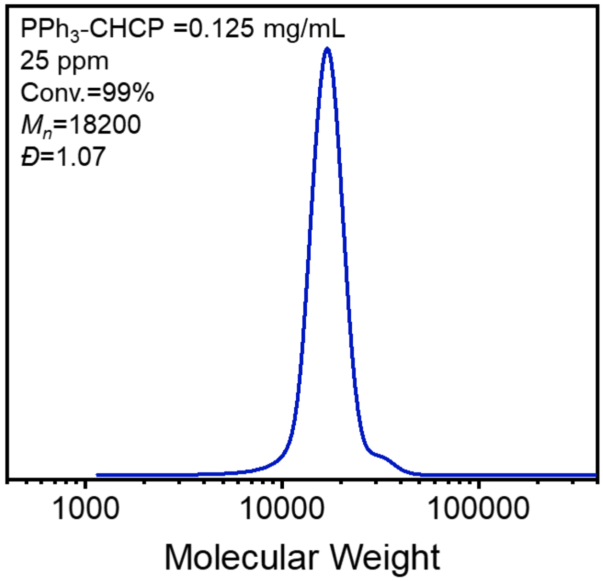

Supplementary Figure 17 | SEC traces of PMA synthesized in the presence of low concentration of both the PPh_3-CHCP and Cu catalyst with Me_6TREN ligand. Reaction conditions: [MA]/[EBiB]/[$CuBr_2$]/[Me_6TREN] = 200/1/0.005/0.12 in DMSO (50 vol%) under green light irradiation (0.9 mW/cm^2), PPh_3-CHCP = 0.125 mg/mL.

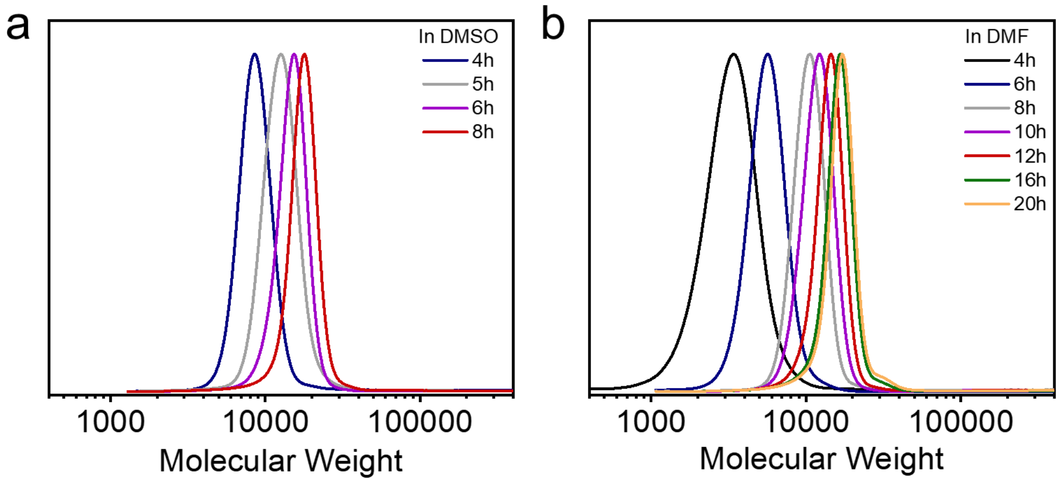

Supplementary Figure 18 | Evolution of SEC traces of PMA synthesized by ATRP using (**a**) 0.5 (in DMSO) and (**b**) 2 mg/mL (in DMF) PPh_3-CHCP photocatalyst in the presence of Me_6TREN ligands respectively. Reaction conditions: [MA]/[EBiB]/[$CuBr_2$]/[Me_6TREN] = 200/1/0.04/x, x = 0.12 or 0.2 in 50 vol % DMSO or DMF, irradiated under green LEDs (0.9 mW/cm^2).

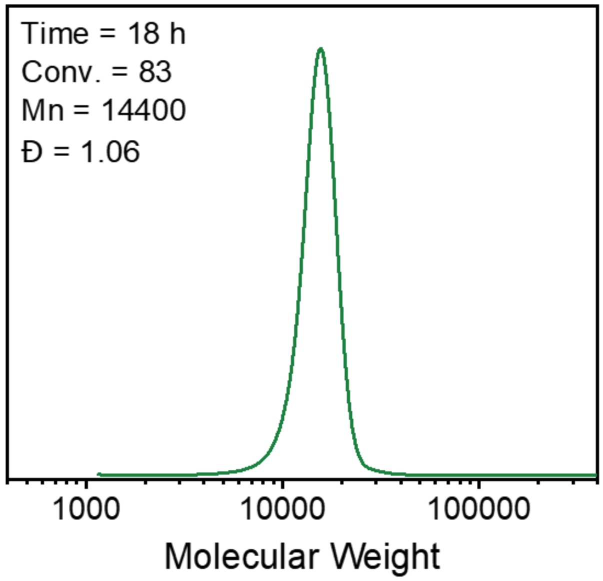

Supplementary Figure 19 | SEC traces of the resulting polymers after intermittent light on/off periods (three cycles of light on/off periods). Reaction conditions: [MA]/[EBiB]/[$CuBr_2$]/[Me_6TREN] = 200/1/0.04/0.2 in 50 vol% DMF, PPh_3-CHCP = 2 mg/mL, irradiated under green light LEDs (0.9 mW/cm^2).

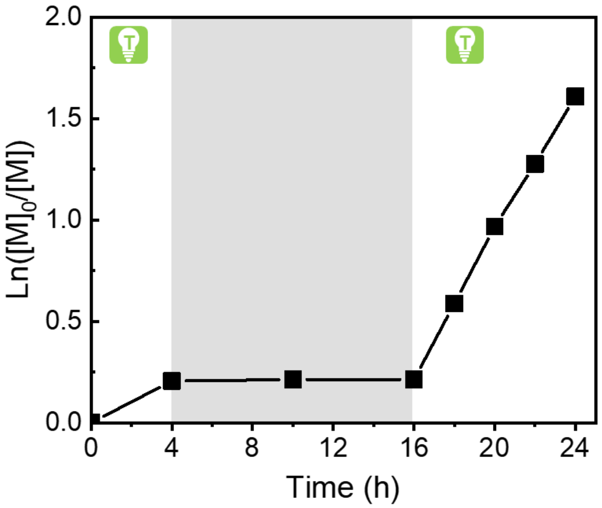

**Supplementary Figure 20 | ** Plot of monomer conversion versus time on the exposure time under green light irradiation (0.9 mW/cm^2) that was switched on and off in the presence of the PPh_3-CHCP photocatalyst. Reaction conditions: [MA]/[EBiB]/[$CuBr_2$]/[Me_6TREN] = 200/1/0.04/0.2 in 50 vol % DMF, PPh_3-CHCP = 2 mg/mL.

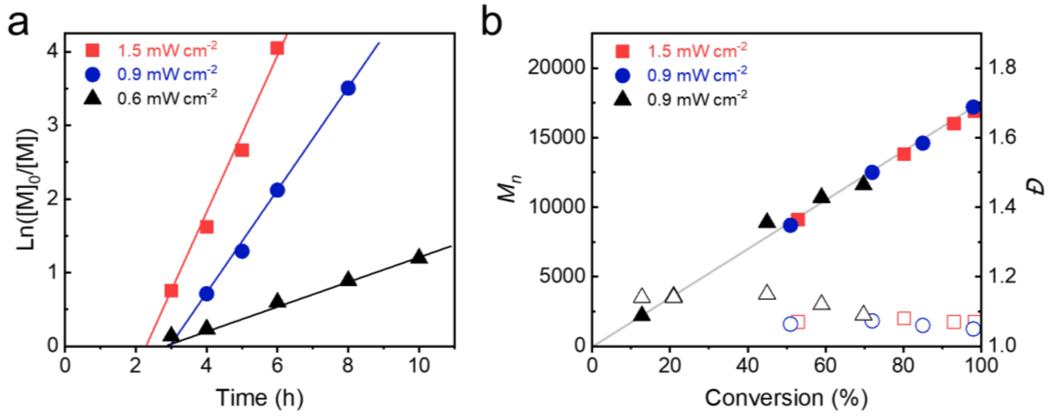

Supplementary Figure 21 | (a) Kinetics and (**b**) evolution of molecular weight (M_n, filled points) and dispersity (Đ, empty points) of the polymers as a function of monomer conversion in the photo-ATRP of MA using 0.5 mg/mL PPh_3-CHCP as a photocatalyst under green light irradiation with different light intensities. Reaction conditions: [MA]/[EBiB]/[$CuBr_2$]/[Me_6TREN]= 200/1/0.04/0.12 in 50 vol% DMSO.

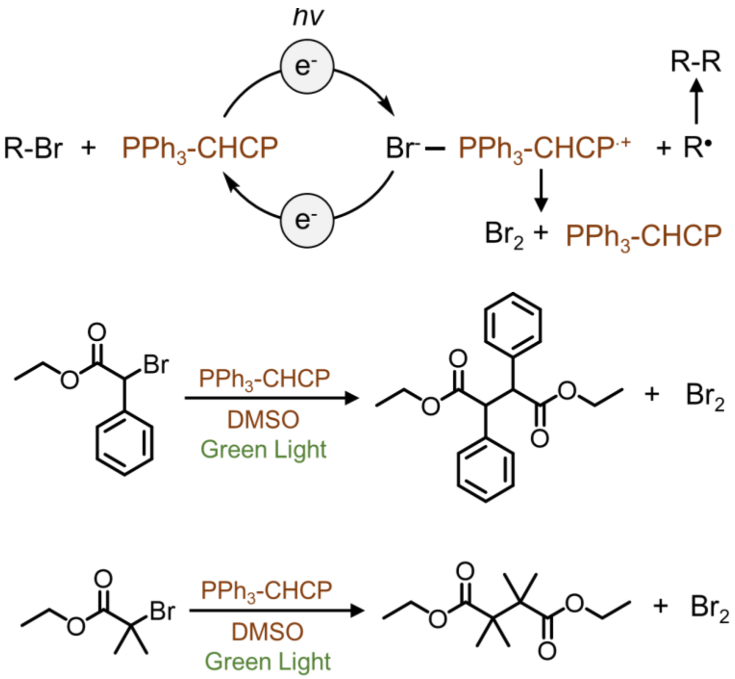

Supplementary Figure 22 | Proposed photoredox cycle of intramolecular electron transfer from PPh_3-CHCP to initiator to generate initial radical.

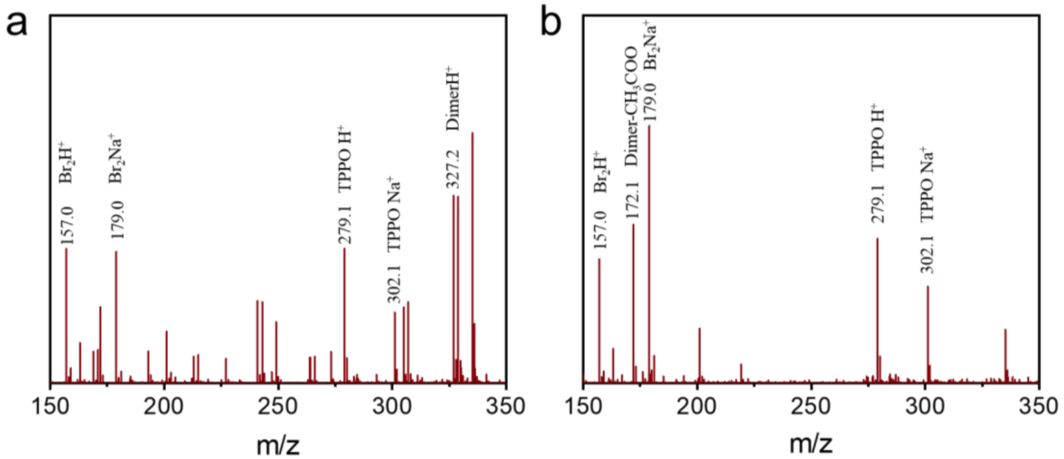

Supplementary Figure 23 | Positive mode ESI mass spectra recorded for EBPA (**a**) and EBiB (**b**) after irradiation under green LEDs (0.9 mW/cm^2) using PPh_3-CHCP as photocatalyst respectively.

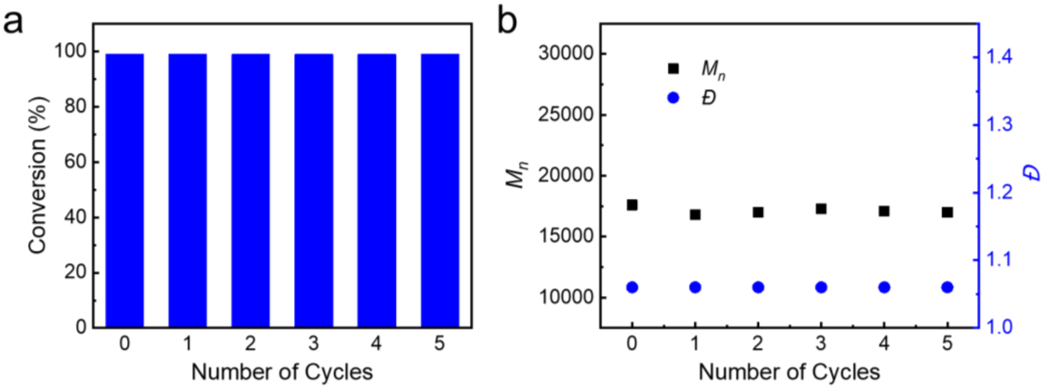

Supplementary Figure 24 | ** Recycling PPh_3-CHCP as a photocatalyst in ATRP of MA showing retention of photocatalytic activity over multiple cycles respectively. (a**) Monomer conversion and (**b**) molecular weight (M_n, squares) and dispersity ($Đ$, circles) of the resulting polymers in recycling experiments. Reaction conditions: [MA]/[EBiB]/[$CuBr_2$]/[Me_6TREN] = 200/1/0.04/0.12 in DMSO (50 vol %) under green light irradiation (0.9 mW/cm^2), PPh_3-CHCP = 0.5 mg/mL.

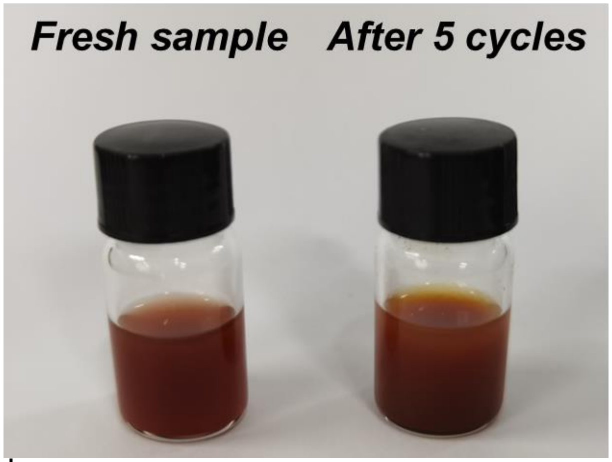

Supplementary Figure 25│Photos of the polymerization solution before (fresh) and after recycling experiments (5 cycles) respectively.

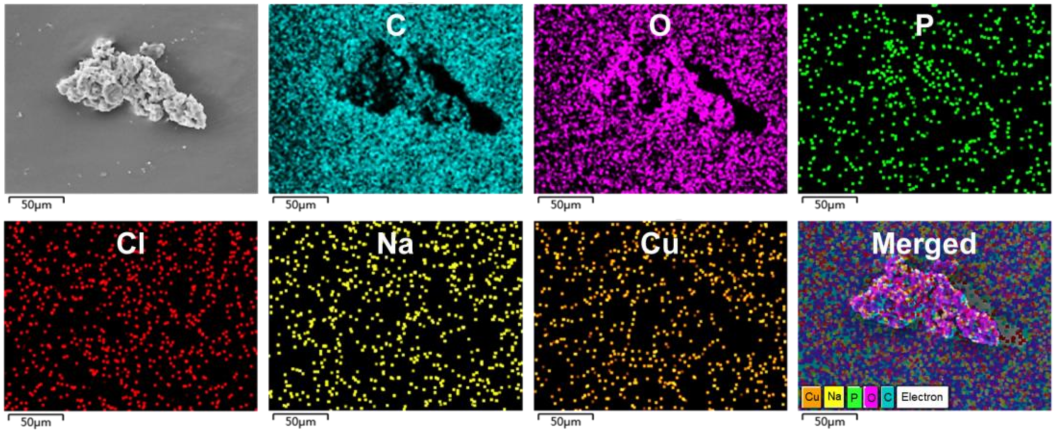

Supplementary Figure 26 | Elemental mapping of recycled PPh_3-CHCP determined by SEM-EDS.

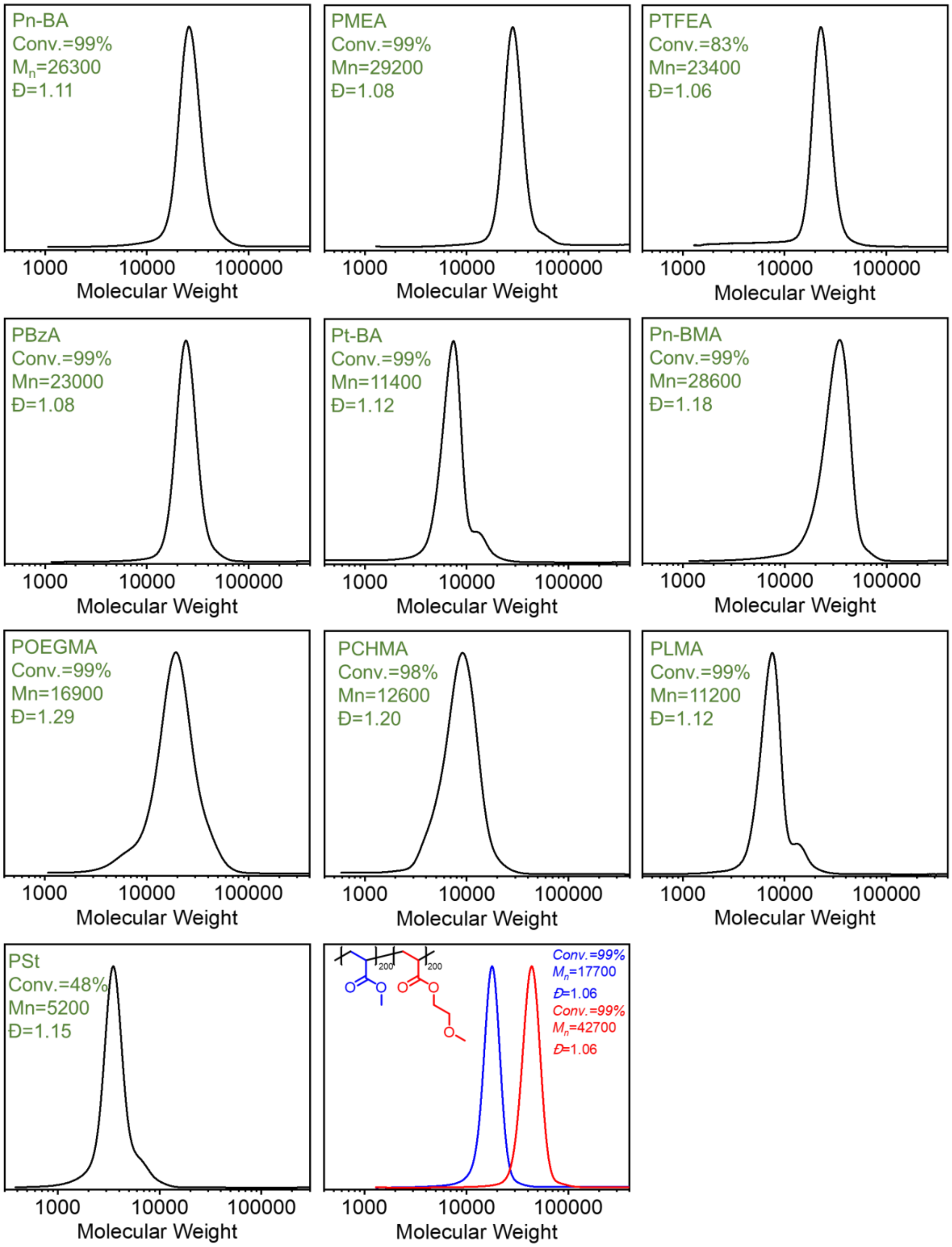

Supplementary Figure 27 | Results of ATRP of acrylate monomers (n-BA, MEA, TFEA, BzA, t-BA, n-BMA, CHMA, LMA, OGEMA, and St) and *in situ* block copolymerization experiments using PPh_3-CHCP photocatalyst. SEC traces of PMA macroinitiator (in blue) and PMA_{200}-*b*-$PMEA_{200}$ block copolymer (in red) upon *in situ* chain extension showing high chain-end fidelity and successful chain extension. Experimental details are provided in the Supplementary Table 4.

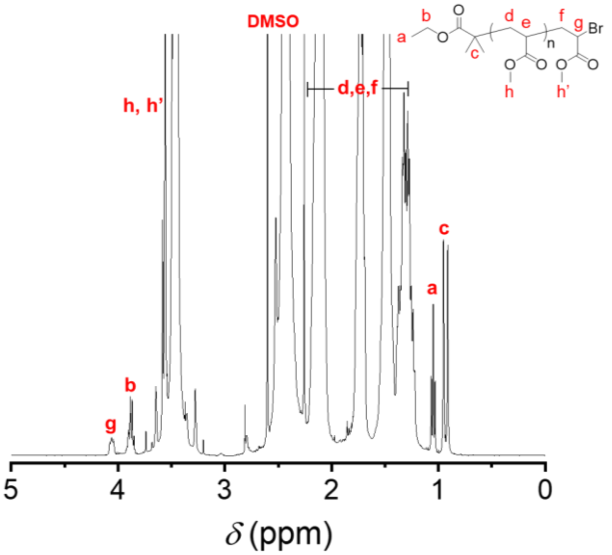

Supplementary Figure 28 | ^{1}H NMR spectrum of final polymer obtained from green light irradiation (0.9 mW/cm^{2}). Integrated ratio of g : c = 0.96 : 6.00. Reaction condition: [MA]/[EBiB]/[$CuBr_2$]/[Me_6TREN]=100/1/0.04/0.12 in 50 vol % DMSO, PPh_3-CHCP = 0.5 mg/mL.

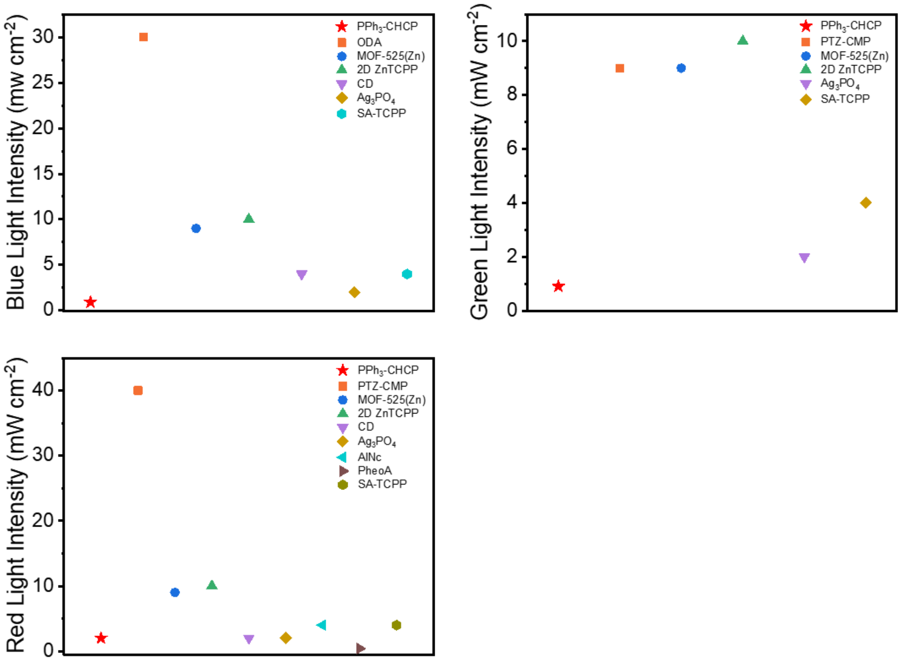

Supplementary Figure 29 | Summary of blue light intensity (*1-6*), green light intensity (*2,3,5-7*), and red light intensity (*2-9*) for various photocatalysts applied in photocatalyzed RDRPs, respectively.

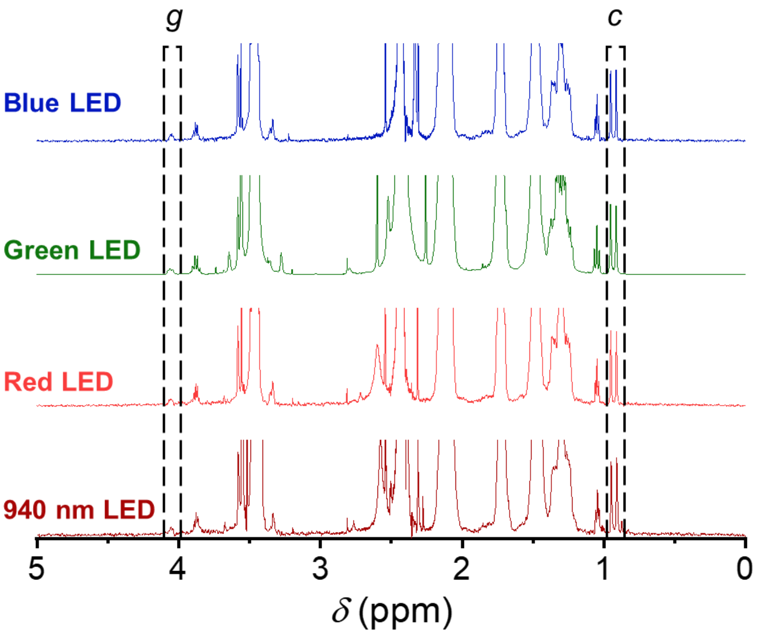

Supplementary Figure 30 | ^{1}H NMR ($CDCl_3$, 600 MHz) spectrum of final polymer obtained from various blue, green, red and 940 nm light irradiation respectively. Experimental details are provided in the Supplementary Table 6.

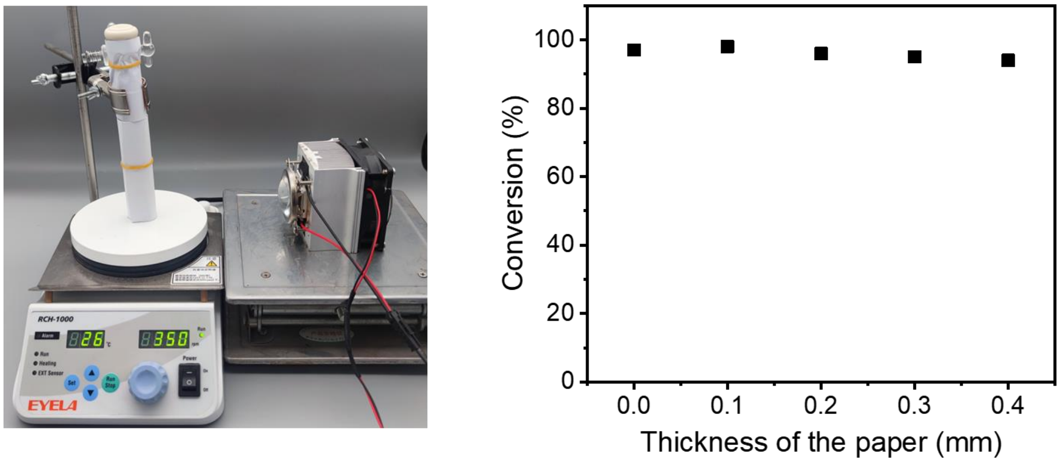

Supplementary Figure 31 | NIR photo ATRP of MA in the presence of opaque paper (left) and the dependence of monomer conversion after 940 nm LED irradiation (30 mW/cm^2) for 6 h on the thickness of the paper (right). Reaction conditions: [MA]/[EBiB]/[$CuBr_2$]/[Me_6TREN] = 200/1/0.02/0.2 in 50 vol % DMSO, PPh_3-CHCP = 2 mg/mL.

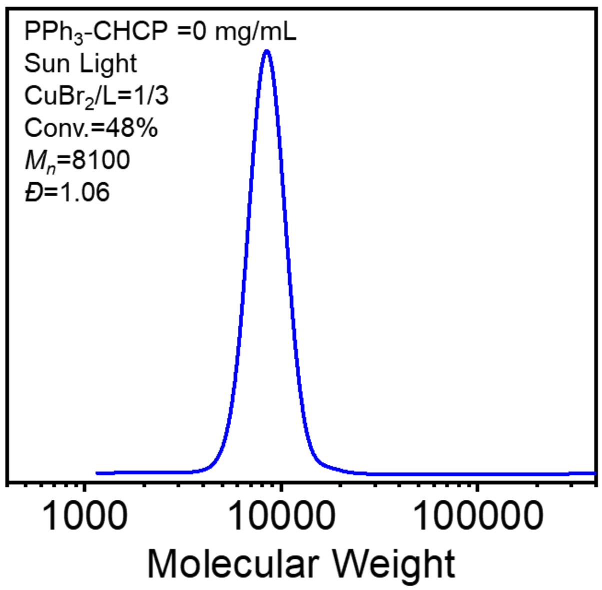

Supplementary Figure 32 | Reaction conditions: [MA]/[EBiB]/[$CuBr_2$]/[Me_6TREN] = 200/1/0.04/0.12 in 50 vol% DMSO under sunlight irradiation for 6 h.

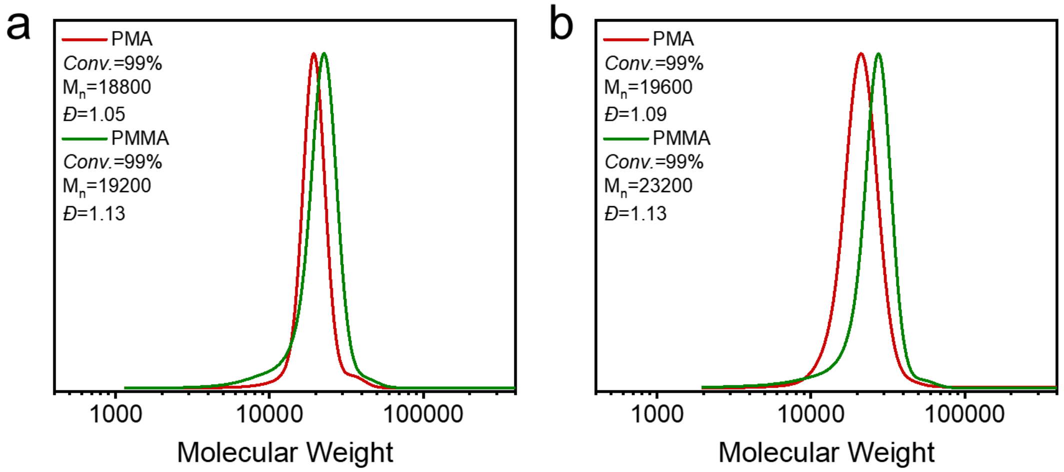

Supplementary Figure 33 | (a) SEC traces of PMA and PMMA synthesized using 0.5 mg/mL PPh_3-CHCP in 50 vol % DMSO under blue light irradiation (0.9 mW/cm^2) in the absence of external deoxygenation respectively. (**b**) SEC traces of PMA and PMMA synthesized using 0.5 mg/mL PPh_3-CHCP in 50 vol % DMSO under sunlight irradiation in the absence of external deoxygenation. Experimental details were provided in the Supplementary Table 12.

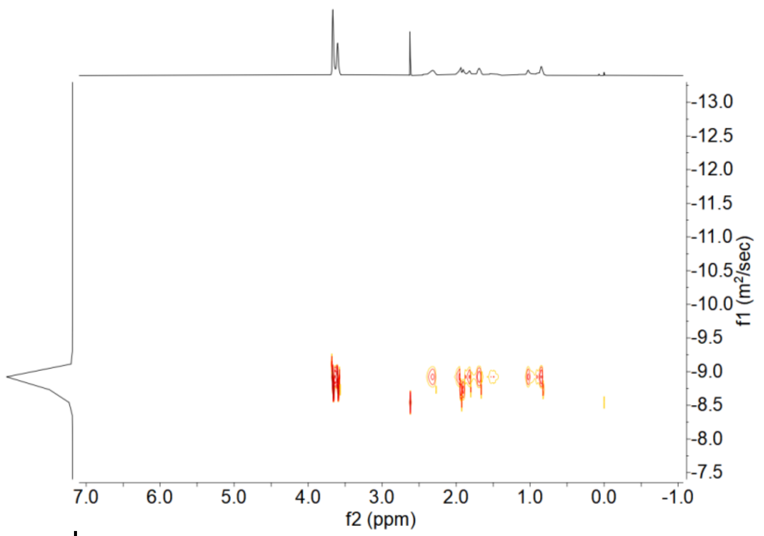

Supplementary Figure 34 | DOSY NMR spectrum of poly(MA_{200}-*b*-MMA_{170}).

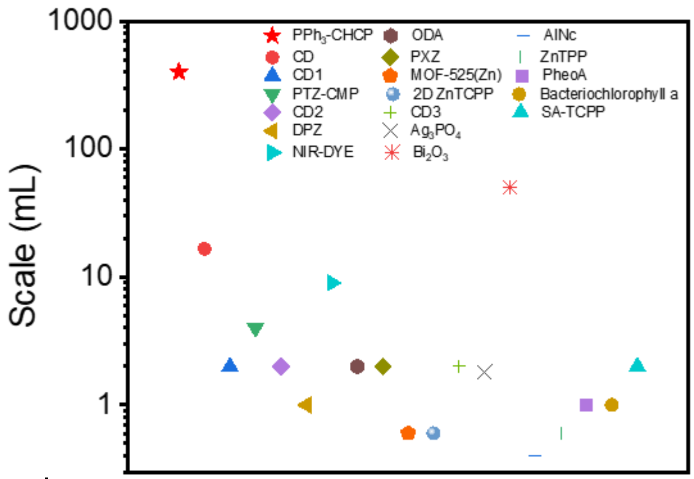

Supplementary Figure 35 | Summary of polymerization scale for various photocatalysts being applied in photo RDRPs (*1-18*).

Details: CD(P-ATRP, 16.6 mL, ref *10*), CD1(P-ATRP, 2 mL, ref *11*), PTZ-CMP(P-ATRP, 4 mL, ref *7*), CD2(P-ATRP, 2 mL, ref *12*), DPZ(P-ATRP, 1 mL, ref *13*), NIY-DYE(P-ATRP, 9 mL, ref *14*), ODA(P-ATRP, 2 mL, ref *1*), PXZ(P-ATRP, 2 mL, ref *15*), MOF-525(Zn)(PET-RAFT, 0.6 mL, ref *2*), 2D ZnTCPP(PET-RAFT, 0.6 mL, ref *3*), CD3(PET-RAFT, 2 mL, ref *4*), Ag_3PO_4(PET-RAFT, 1.8 mL, ref *5*), Bi_2O_3 (PET-RAFT, 50 mL, ref *16*), AINc(PET-RAFT, 0.4 mL, ref *8*), ZnTPP(PET-RAFT, 0.6 mL, ref *17*), PheoA(PET-RAFT, 1 mL, ref *9*), Bacteriochlorophyll *α* (PET-RAFT, 1 mL, ref *18*), SA-TCPP(PET-RAFT, 2 mL, ref *6*).

λ_{ex} [nm][a]	332	490
λ_{em} [nm][b]	480	580
τ_s [ns][c]	2.63	3.76
Φ [%][d]	24.53	11.70

Supplementary Table 1│Fluorescent properties of PPh_3-CHCP in DMSO at an excitation wavelength of 332 and 490 nm respectively. [a] Excitation wavelength. [b] Emission wavelength. [c] Fluorescence lifetime. [d] Quantum yield.

entry	Photocatalyst	Conv (%)	$M_{n,th}$	M_n	$Đ$
1	2 mg/mL PPh_3-HCP	19	3500	1200	1.17
2	2 mg/mL PPh_3-CHCP	99	17300	17000	1.05

Supplementary Table 2 | Results of control experiments in polymerization of MA using PPh_3-HCP and PPh_3-CHCP photocatalyst under green light irradiation respectively. Reaction conditions: [MA]/[EBiB]/[$CuBr_2$]/[Me_6TREN] = 200/1/0.04/0.2 in 50 vol% DMF under green light irradiation (0.9 mW/cm^2) for 24 h.

	PPh_3-HCP	PPh_3-CHCP
Zeta Potential (mV)	-32.9	-43
Conductivity (mS/cm)	0.0375	0.076

Supplementary Table 3 | Zeta Potential and conductivity of PPh_3-HCP and PPh_3-CHCP.

entry	Monomer	Initiator	Ligand	$CuBr_2$/ L	PPh_3-CHCP (mg/mL)	Solvent	Time (h)	Conv (%)	$M_{n,th}$	M_n	*Đ*
1	n-BA	EBiB	Me_6TREN	1/5	2	MeCN	24	99	25700	26300	1.11
2	MEA	EBiB	Me_6TREN	1/3	0.25	DMSO	8	99	26100	29200	1.08
3	TFEA	EBiB	Me_6TREN	1/3	0.5	DMSO	16	83	25700	23400	1.06
4	BzA	EBiB	Me_6TREN	1/3	0.5	DMSO	16	99	31300	23000	1.08
5	t-BA	Br-PEG-Br	Me_6TREN	1/5	2	MeCN	24	99	12000	11400	1.12
6	n-BMA	EBPA	TPMA	1/5	0.5	MeCN	48	99	29000	28600	1.18
7	CHMA	Br-PEG-Br	Me_6TREN	1/5	2	DMF	24	98	14400	12600	1.20
8	LMA	Br-PEG-Br	Me_6TREN	1/5	2	DMF	24	99	9300	11200	1.12
9	OEGMA	HBiB	TPMA	1/5	0.5	Water	10	99	15200	16900	1.29
10[a]	St	EBPA	TPMA /Me_6TREN	1/2.5/2.5	0.5	DMF /DMSO	24	48	5000	5200	1.15

Supplementary Table 4 | Monomer scope of PPh_3-CHCP photocatalyzed ATRPs. Polymerizations were performed according to the above general polymerization procedure using various initiator and irradiated under green LEDs (0.9 mW/cm^2) (entry 1-9) or blue LEDs (2 mW/cm^2) (entry 10). Reaction conditions: In entry 1-4, [M]/[EBiB]/[$CuBr_2$]/[Me_6TREN] = 200/1/0.04/x in 50 vol% solvent (x=0.12 or 0.2). [t-BA]/[Br-PEG-Br]/[$CuBr_2$]/[Me_6TREN] = 60/0.5/0.04/0.2 in 67 vol% MeCN. [n-BMA]/[EBPA]/[$CuBr_2$]/[TPMA]/[TEOA] = 200/1/0.04/0.2/0.6 in 50 vol% MeCN. [CHMA]/[Br-PEG-Br]/[$CuBr_2$]/[Me_6TREN] = 60/0.5/0.04/0.2 in 67 vol% DMF. [LMA]/[Br-PEG-Br]/[$CuBr_2$]/[Me_6TREN] = 20/0.5/0.04/0.2 in 67 vol% DMF. [OEGMA]/[HBiB]/[$CuBr_2$]/[TPMA]/[NaBr]/[TEOA] = 50/1/0.04/0.12/0.4/0.6 in 75 vol% water. [a][St]/[EBPA]/[$CuBr_2$]/[TPMA]/[Me_6TREN]/[TEOA] = 100/1/0.04/0.1/0.1/0.6 in 50 vol% solvent (V_{DMF}/V_{DMSO} = 1/1). Considering the low reactivity of styrene monomer, polymerization was conducted at temperature of 35°C.

entry	Monomer	Light Source	$CuBr_2$/L	PPh_3-CHCP (mg/mL)	Time (h)	Conv (%)	$M_{n,th}$	M_n	$Đ$
1	MA	Blue-0.9 mW/cm^2	1/3	0.5	3	99	17300	16600	1.07
2	MA	Green-0.9 mW/cm^2	1/3	0.5	8	99	17300	17600	1.06
3	MA	Orange-2.0 mW/cm^2	1/5	1	12	99	17300	17900	1.06
4	MA	Red-2.0 mW/cm^2	1/5	1	12	99	17300	18000	1.07
5	MA	White-0.9 mW/cm^2	1/3	0.5	12	99	17300	16900	1.07
6	MA	730 nm-10 mW/cm^2	1/5	1	16	99	17300	18100	1.08
7	MA	760 nm-10 mW/cm^2	1/5	1	12	99	17300	18100	1.07
8	MA	800 nm-10 mW/cm^2	1/5	1	12	99	17300	16600	1.09
9	MA	850 nm-15 mW/cm^2	1/5	1	16	99	17300	18400	1.08
10	MA	940 nm-15 mW/cm^2	1/5	1	12	99	17300	17100	1.06
11	MA	Sun-100 mW/cm^2	1/3	0.5	6	99	17300	18600	1.06
12*[a]	MA	Sun-100 mW/cm^2	1/3	0.5	6	99	17300	17300	1.08
13	MMA	Blue-0.9 mW/cm^2	1/5	0.5	8	99	20100	25600	1.08
14	MMA	Green-0.9 mW/cm^2	1/5	0.5	16	99	20100	19600	1.12
15	MMA	Orange-2.0 mW/cm^2	1/5	1	24	99	20100	24200	1.11
16	MMA	Red-2.0 mW/cm^2	1/5	1	36	99	20100	25500	1.12
17	MMA	White-0.9 mW/cm^2	1/5	0.5	12	99	20100	24200	1.10
18	MMA	730 nm-10 mW/cm^2	1/5	1	28	99	20100	24000	1.11
19	MMA	760 nm-10 mW/cm^2	1/5	1	28	99	20100	20600	1.10
20	MMA	800 nm-10 mW/cm^2	1/5	1	48	99	20100	16600	1.10
21	MMA	850 nm-15 mW/cm^2	1/5	1	48	99	20100	17000	1.11
22	MMA	940 nm-15 mW/cm^2	1/5	1	44	99	20100	19400	1.11
23	MMA	Sun-100 mW/cm^2	1/5	0.5	6	99	20100	19700	1.12
24*[a]	MMA	Sun-100 mW/cm^2	1/5	0.5	6	99	20100	22700	1.15

Supplementary Table 5 | Polymerization of MMA and MA using PPh_3-CHCP photocatalyst under blue, green, orange, red, white, 730 nm, 760 nm, 800 nm, 850 nm, 940 nm, and sunlight irradiation respectively. Reaction conditions: [MA]/[EBiB]/[$CuBr_2$]/[Me_6TREN] = 200/1/0.04/x in 50 vol% DMSO (x = 0.12 or 0.2). [MMA]/[EBPA]/[$CuBr_2$]/[TPMA] = 200/1/0.04/0.2 in 50 vol% DMSO, TEOA (0.6 equiv relative to EBPA) was used as the electron donor in the presence of TPMA. [a]Without prior deoxygenation process. Asterisk indicated 100 mL scale of monomer. Experimental details are provided in the supplementary materials.

entry	Light Source	Time (h)	Conv (%)	$M_{n,NMR}$	$M_{n,SEC}$	$Đ$	g:c
1	Blue-0.9 mW/cm^2	3	99	7800	7500	1.07	0.99:6
2	Green-0.9 mW/cm^2	10	98	8400	8200	1.06	0.96:6
3	Red-2 mW/cm^2	12	98	7600	8500	1.06	0.99:6
4	940 nm-15 mW/cm^2	12	96	8000	8000	1.06	0.97:6

Supplementary Table 6 | The effect on wavelength on the end group in photo-mediated polymerization of MA. Reaction condition: [MA]/[EBiB]/[$CuBr_2$]/[Me_6TREN] = 100/1/0.04/x in 50 vol% DMSO (x = 0.12 and 0.5 mg/mL PPh_3-CHCP for blue and green light irradiation, x = 0.2 and 1 mg/mL PPh_3-CHCP for red and 940 nm light irradiation).

entry	Barrier [mm]	Time (h)	Conv (%)	$M_{n,th}$	M_n	$Đ$
1	-	2	97	16900	17100	1.22
2	0.1	6	98	16700	17900	1.15
3	0.2	6	96	16900	17100	1.14
4	0.3	6	95	16600	20000	1.20
5	0.4	6	94	16400	17900	1.28

Supplementary Table 7 | NIR photocatalyzed polymerization of MA in the presence of translucent barrier. Reaction conditions: [MA]/[EBiB]/[$CuBr_2$]/[Me_6TREN] = 200/1/0.02/0.2 (0.02 equiv with respect to initiator corresponding to 100 ppm with respect to monomer) in 50 vol % DMSO under 940 nm light irradiation (30 mW/cm^2), PPh_3-CHCP = 2 mg/mL. Translucent barrier is A4 paper.

Material	Method	Light Source	Monomer	Conv (%)	Time (h)	*Ref.*
PPh_3-CHCP	P-ATRP	940 nm-15 mW/cm^2	MA	99	12	This Work
			MMA	99	44	
NIR-Dye	P-ATRP	790 nm-100 mW/cm^2	MMA	59	48	*14*
$UCNP@SiO_2@N$-CDs	P-ATRP	980 nm-1.5 W/cm^2	MMA	46	22	*21*
			HEA	66	5	
β-$NaYF^4$:30% Yb^{3+}, 1% Tm^{3+}	P-ATRP	980 nm-4 W/cm^2	MA	67	24	*23*
			MMA	20		
			AN	60	12	
ZnTtBAzP	PET-RAFT	780 nm-10 W/cm^2	MA	80	3.3	*19*
AlNc	PET-RAFT	850 nm-20 mW/cm^2	MA	82	0.5	*8*
Bacteriochlorophyll *α*	PET-RAFT	780 nm-20 mW/cm^2	MMA	44	16	*18*
Ag_3PO_4	PET-RAFT	940 nm-16 mW/cm^2	BzA	99	18.8	*5*
SA-TCPP	PET-RAFT	850 nm-4.0 mW/cm^2	DMA	29.8	64	*6*
AlPc	PET-RAFT	780 nm-6.2 mW/cm^2	MA	66	6	*22*
RTPP	PET-RAFT	740 nm-66 mW/cm^2	MMA	89	12	*20*
			BA	30	6	
$CsPbBr_3$ NCs	PET-RAFT	800 nm-3 W/cm^2	MA	60.2	11	*24*
$CsPbI_3$@PCN-222	PET-RAFT	850 nm-50 mW/cm^2	MMA	99	10	*25*
$NaYF_4$:Yb/Tm	PET-RAFT	980 nm-2 W/cm^2	n-BA	100	5	*26*
β-$NaYF_4$:Yb/Tm	SI-PET-RAFT	980 nm-1.5 W/cm^2	t-BA	78	24	*27*
Au NR	PET-RAFT	980 nm-0.5 mW/cm^2	MMA	16.5	72	*28*
Au/g-C_3N_4	PET-RAFT	740 nm-1.5 mW/cm^2	MMA	18.1	20	*29*

Supplementary Table 8 | Summary of NIR photo RDRP.

Material	Method	Light Source	Monomer	Conv (%)	Time (h)	Barrier	*Ref.*
PPh_3-CHCP	P-ATRP	940 nm-30 mW/cm^2	MA	94	6	0.4 mm A4 paper	This Work
β-$NaYF^4$:30% Yb^{3+}, 1% Tm^{3+}	P-ATRP	980 nm-4 W/cm^2	MMA	64	36	0.2 mm A4 paper	*23*
				88		1.2 mm pig skin	
UCNP@SiO_2@N-CDs	P-ATRP	980 nm-1.5 W/cm^2	MMA	45	24	1.2 mm pig skin	*21*
Bacteriochlorophyll *α*	PET-RAFT	850 nm-40 mW/cm^2	MMA	24	20	0.2 mm A4 paper	*18*
Ag_3PO_4	PET-RAFT	780 nm-6 mW/cm^2	BzA	88	21	0.1 mm A4 paper	*5*
AlNc	PET-RAFT	850 nm-100 mW/cm^2	MA	70	1.25	0.2 mm A4 paper	*8*
				73	1.3	5.0 mm pig skin	
RTPP	PET-RAFT	740 nm-66 mW/cm^2	MMA	50	15	7 mm pig skin	*20*

Supplementary Table 9 | Summary of NIR penetration photo RDRP with material barriers.

entry	[M]/[I]/[$CuBr_2$]/[L]	Conv (%)	$M_{n,th}$	M_n	$Đ$
1	[MA]/[EBiB]/[$CuBr_2$]/[Me_6TREN] = 200/1/0.04/0.12	48	8500	8100	1.06
2[a]	[MMA]/[EBPA]/[$CuBr_2$]/[TPMA] = 200/1/0.04/0.2	0	-	-	-

Supplementary Table 10 | Results of control experiments in polymerization of MA and MMA without using PPh_3-CHCP under sunlight irradiation respectively. Polymerizations were performed in 50 vol% DMSO under sunlight irradiation for 6 h. [a]TEOA (0.6 equiv relative to EBPA) was used as the electron donor in the presence of TPMA.

entry	Light	Time (h)	Conv (%)	$M_{n,th}$	M_n	$Đ$
1	Blue	3	0	-	-	-
2	Green	8	0	-	-	-

Supplementary Table 11 | Results of control experiments in polymerization of MA without using PPh_3-CHCP under blue and green light irradiation respectively. Reaction conditions: [M]/[I]/[$CuBr_2$]/[Me_6TREN] = 200/1/0.04/0.12 in 50 vol% DMSO under blue or green light irradiation (0.9 mW/cm^2) for preset time interval.

entry	Light	[M]/[I]/[$CuBr_2$]/[L]	Time (h)	Conv (%)	$M_{n,th}$	M_n	$Đ$
1	Blue	[MA]/[EBiB]/[$CuBr_2$]/[Me_6TREN] = 200/1/0.04/0.12	3	0	-	-	-
2[a]	Blue	[MMA]/[EBPA]/[$CuBr_2$]/[TPMA] = 200/1/0.04/0.2	8	-	-	7000	1.10
3	Green	[MA]/[EBiB]/[$CuBr_2$]/[Me_6TREN] = 200/1/0.04/0.12	8	0	-	-	-
4[a]	Green	[MMA]/[EBPA]/[$CuBr_2$]/[TPMA] = 200/1/0.04/0.2	16	29	6000	6200	1.10

Supplementary Table 12 | Results of control experiments in polymerization of MA and MMA using 0.5 mg/mL PPh_3-CHCP under blue light or sunlight irradiation in the absence of external deoxygenation. [a]The polymerizations were performed in 50 vol% DMSO under blue light irradiation (0.9 mW/cm^2). [b]TEOA (0.6 equiv relative to EBPA) was used as the electron donor in the presence of TPMA. [c]The polymerizations were performed in 50 vol% DMSO under sunlight irradiation.

Supplementary References

1. Ma, Q. et al. Metal-free atom transfer radical polymerization with ppm catalyst loading under sunlight. *Nat. Commun.* **12**, 429 (2021).
2. Zhang, L. et al. 2D porphyrinic metal-organic framework nanosheets as multidimensional photocatalysts for functional materials. *Angew. Chem. Int. Ed.* **60**, 22664-22671 (2021).
3. Zhang, L. et al. Porphyrinic zirconium metal-organic frameworks (MOFs) as heterogeneous photocatalysts for PET-RAFT polymerization and stereolithography. *Angew. Chem. Int. Ed.* **60**, 5489-5496 (2021).
4. Jiang, J. Ye, G. Wang, Z. Lu, Y. Chen, J. & Matyjaszewski, K. Heteroatom-doped carbon dots (CDs) as a class of metal-free photocatalysts for PET-RAFT polymerization under visible light and sunlight. *Angew. Chem., Int. Ed.* **57**, 12037-12042(2018).
5. Jiang, J. et al. Localized surface plasmon resonance meets controlled/living radical polymerization: an adaptable strategy for broadband light-regulated macromolecular synthesis. *Angew. Chem. Int. Ed.* **58**, 12096-12101 (2019).
6. Allison-Logan, S. et al. From UV to NIR: a full-spectrum metal-free photocatalyst for efficient polymer synthesis in aqueous conditions. *Angew. Chem. Int. Ed.* **59**, 21392-21396 (2020).
7. Dadashi-Silab, S. et al. Conjugated cross-linked phenothiazines as green or red light heterogeneous photocatalysts for copper-catalyzed atom transfer radical polymerization. *J. Am. Chem. Soc.* **143**, 9630-9638 (2021).
8. Wu, Z. Jung, K. & Boyer, C. Effective utilization of NIR wavelengths for photo-controlled polymerization: penetration through thick barriers and parallel solar syntheses. *Angew. Chem. Int. Ed.* **59**, 2013-2017 (2020).
9. Xu, J. Shanmugam, S. Fu, C. Aguey-Zinsou, K. F. & Boyer, C. Selective photoactivation: from a single unit monomer insertion reaction to controlled polymer architectures. *J. Am. Chem. Soc.* **138**, 3094-3106 (2016).
10. Kütahya, C. et al. Carbon dots as a promising green photocatalyst for free radical and ATRP-based radical photopolymerization with blue LEDs. *Angew. Chem. Int. Ed.* **59**, 3166-3171 (2020).
11. Kutahya, C. et al. Distinct sustainable carbon nanodots enable free radical photopolymerization, photo-ATRP and photo-CuAAC chemistry. *Angew. Chem. Int. Ed.* **60**, 10983-10991 (2021).
12. Qiao, L. et al. Ultrafast visible-light-induced ATRP in aqueous media with carbon quantum dots as the catalyst and its application for 3D printing. *J. Am. Chem. Soc.* **144**, 9817-9826 (2022).

13. Theriot, J. C. et al. Organocatalyzed atom transfer radical polymerization driven by visible light. *Science* **352**, 1082-1086 (2016).
14. Kütahya, C. Schmitz, C. Strehmel, V. Yagci, Y. & Strehmel, B. Near-infrared sensitized photoinduced atom-transfer radical polymerization (ATRP) with a copper (II) catalyst concentration in the ppm range. *Angew. Chem. Int. Ed.* **57**, 7898-7902 (2018).
15. McCarthy, B. G. et al. Structure-property relationships for tailoring phenoxazines as reducing photoredox catalysts. *J. Am. Chem. Soc.* **140**, 5088-5101 (2018).
16. Hakobyan, K. Gegenhuber, T. McErlean, C. S. P. & Mullner, M. Visible-light-driven MADIX polymerisation *via* a reusable, low-cost, and non-toxic bismuth oxide photocatalyst. *Angew. Chem. Int. Ed.* **58**, 1828-1832 (2019).
17. Shanmugam, S. Xu, J. & Boyer, C. Exploiting metalloporphyrins for selective living radical polymerization tunable over visible wavelengths. *J. Am. Chem. Soc.* **137**, 9174-9185 (2015).
18. Shanmugam, S. Xu, J. & Boyer, C. Light-regulated polymerization under near-infrared/far-red irradiation catalyzed by bacteriochlorophyll α. *Angew. Chem. Int. Ed.* **128**, 1048-1052 (2016).
19. Wu, C. Jung, K. Ma, Y. Liu, W. & Boyer, C. Unravelling an oxygen-mediated reductive quenching pathway for photopolymerisation under long wavelengths. *Nat. Commun.* **12**, 478(2021).
20. Cao, H. et al. Far-red light-induced reversible addition-fragmentation chain transfer polymerization using a man-made bacteriochlorin. *ACS Macro Lett.* **8**, 616-622 (2019).
21. Qiao, X. et al. Simple full-spectrum heterogeneous photocatalyst for photo-induced atom transfer radical polymerization (ATRP) under UV/vis/NIR and its application for the preparation of dual mode curing injectable photoluminescence hydrogel. *ACS Appl. Mater. Interfaces* **14**, 21555-21563 (2022).
22. Corrigan, N. Xu, J. T. & Boyer, C. A Photoinitiation system for conventional and controlled radical polymerization at visible and NIR wavelengths. *Macromolecules* **49**, 3274-3285 (2016).
23. Zhang, W. et al. Atom transfer radical polymerization driven by near-infrared light with recyclable upconversion nanoparticles. *Macromolecules* **53**, 4678-4684 (2020).
24. Zhu, Y. Liu, Y. Miller, K. A. Zhu, H. & Egap, E. Lead halide perovskite nanocrystals as photocatalysts for PET-RAFT polymerization under visible and near-infrared irradiation. *ACS Macro Lett.* **9**, 725-730 (2020).

25. Xia, Z. N. Shi, B. F. Zhu, W. J. Xiao, Y. & Lu, C. L. Binary hybridization strategy toward stable porphyrinic Zr-MOF encapsulated perovskites as high-performance heterogeneous photocatalysts for red to NIR light-induced PET-RAFT polymerization. *Adv. Funct. Mater.* **32**, 2207655 (2022).
26. Ding, C. et al. Platform of near-infrared light-induced reversible deactivation radical polymerization: upconversion nanoparticles as internal light sources. *Polym. Chem.* **7**, 7370-7374 (2016).
27. Hu, L. J. et al. The *in situ* "grafting from" approach for the synthesis of polymer brushes on upconversion nanoparticles via NIR-mediated RAFT polymerization. *Polym. Chem.* **12**, 545 (2021).
28. Zhang, J. et al. From 0-dimension to 1-dimensions: Au nanocrystals as versatile plasmonic photocatalyst for broadband light induced RAFT polymerization. *Polym. Chem.* **12**, 2439-2446 (2021).
29. Li, M. et al. Dual enhancement of carrier generation and migration on Au/g-C_3N_4 photocatalysts for highly-efficient broadband PET-RAFT polymerization. *Polym. Chem.* **13**, 1022-1030 (2022).
